# Safety and risks of CBD oils purchased online: unveiling uncertain quality and vague health claims

**DOI:** 10.3389/fphar.2023.1273540

**Published:** 2023-12-14

**Authors:** Róbert György Vida, Lilien Victoria Strauss, Ákos Bajtel, Tivadar Kiss, Dezső Csupor, András Fittler

**Affiliations:** ^1^ Department of Pharmaceutics, Faculty of Pharmacy, University of Pécs, Pécs, Hungary; ^2^ Institute of Pharmacognosy, Faculty of Pharmacy, University of Szeged, Szeged, Hungary; ^3^ Institute of Clinical Pharmacy, Faculty of Pharmacy, University of Szeged, Szeged, Hungary; ^4^ Institute for Translational Medicine, Medical School, University of Pécs, Pécs, Hungary

**Keywords:** cannabidiol oil, cannabidiol, consumer product safety, online medicinal products, product labeling, quality, test purchase

## Abstract

**Introduction:** The unmet need for highly effective, naturally derived products with minimal side effects results in the over-popularity of ever-newer medicinal plants. In the middle of 2010, products containing cannabidiol (CBD), one of the special metabolites of *Cannabis sativa*, started to gain popularity. For consumers and healthcare providers alike, the legal context surrounding the marketing of CBD products is not entirely clear, and the safety of using some products is in doubt. Companies in the online medicinal product market profit from the confusion around CBD oils.

**Methods:** In our study, we employed a complex method known as risk-based safety mapping of the online pharmaceutical market, which included health claim content analysis of online stores, test purchases, and labeling and quantitative analysis of the CBD content.

**Results:** There were discovered 16 online retailers selling an average of 2–7 goods and CBD oils with a concentration of 3%–5% (30–50 mg/mL) CBD. The majority (n/N = 10/16, 62.5%) displayed potential health-related benefits indirectly on their website, and in the case of one web shop (n/N = 1/16, 6.3%), we detected COVID-19-related use. Altogether, 30 types of purported “indications” were collected. A total of 12 CBD oil products were test-purchased from online retailers in December 2020. Upon evaluating the packaging and product information, we noticed that three products (n/N = 3/12, 25%) lacked instructions on use, hence increasing the risk of inappropriate application and dosing. The cannabidiol content was quantified using UHPLC. The measured CBD concentrations of the products ranged from 19.58 mg/mL to 54.09 mg/mL (mean 35.51 mg/mL, median 30.63 mg/mL, and SD ± 12.57 mg/mL). One (8.33%) product was underlabeled, five (41.67%) were over-labeled, and only every second product (50%) was appropriately labeled based on the quantitative assessment of CBD concentration.

**Discussion:** Further research and quality control are necessary to establish the regulatory context of the usage and classification of CBD and other cannabinoids in nonmedicinal products (e.g., food supplements), as authorities and policymakers worldwide struggle with the uncertainties surrounding CBD products.

## 1 Introduction

Phytocannabinoids are characteristic metabolites of *Cannabis sativa* L. Approximately 200 cannabinoids have been discovered from this species, each with unique therapeutic and/or abuse potential. These bioactive terpenoids have recently been discovered in *Rhododendron* and *Radula* species, as well as in certain legumes and fungi (Gülck and Møller, 2020; Govindarajan et al., 2023; Hesami et al., 2023; Sainz Martinez et al., 2023). Cannabinoids are insoluble in water but soluble in alcohols, non-polar organic solvents (e.g., ether and hexane), and lipids. That is why the first products encountered in 2015 were mostly oil-based (Messina et al., 2015; Thomas and ElSohly, 2016). The revival of plant-based products and the steady shift in attitude surrounding *Cannabis sativa* L. consumption created an opportunity for cannabidiol (CBD) products, which inundated offline and online health markets (Hazekamp, 2018). In 2018, the “Farm Bill,” known as the Agricultural Improvement Act, permitted hemp-derived products to be treated as agricultural products, with a restriction of maximum 0.3% (w/w) tetrahydrocannabinol (THC) content by dry weight, and removed these products from the list of controlled substances under the supervision of the Drug Enforcement Administration (DEA) in the United States (Drug Enforcement Administration, 2018; Dabrowska et al., 2020). The opioid epidemic, which raised demand for non-opioid analgesic alternatives, also contributed to the emergence of the *Cannabis* and CBD product markets (Olfson et al., 2018).

The rise of CBD products began in 2014–2015, with the introduction of numerous food supplements not only in the United States but also in Europe. Since its distribution as food is incompatible with the novel food regulation (European Parliament, 2015), it can no longer be sold as a food supplement or conventional food ingredient. As a result, manufacturers resorted to other non-medical health product categories, such as medical devices or cosmetics. This legal ambiguity, along with the increase in demand over the past decade, has spawned a multibillion-dollar global market (Sarma et al., 2020; Bhamra et al., 2021; Miller et al., 2022; Lachenmeier et al., 2023).

Given that these plant-based products were initially marketed as food supplements, it is not surprising that health claims for CBD products have appeared. Regulatory agencies identified several regulatory violations, including the use of medical claims, and such incidents are still frequent among these products (Amann et al., 2022; Spindle et al., 2022). The potential long-term adverse health effects, including liver toxicity, possible harm to the male reproductive system, and the increased risk of drug interactions, also call for stricter control of CBD-containing products and emphasize the dangers of their uncontrolled use as food supplements (Huestis et al., 2019).

Globally, there are five cannabinoid-containing products authorized by regulatory agencies as medicine: cannabidiol (Epidiolex^®^ or Epidyolex^®^), dronabinol/delta-tetrahydro-cannabinol (Marinol^®^, Syndros^®^, Reduvo^®^, and Adversa^®^), nabilone (Cesamet^®^ and Canemes^®^), and nabiximols (CBD:THC = 1:1; Sativex^®^). The Summary of Product Characteristics (SmPC) of these products may help identify the potential adverse health consequences of unregulated CBD oil and specific patient populations that will not benefit from their use (Kalant and Porath-Waller, 2016; Bajtel et al., 2021; Schlag et al., 2022).

National regulatory agencies are responding differently to the unresolved issue of CBD products because the current regulatory frameworks are incapable of mitigating these risks, and new approaches are required. The Hungarian National Institute of Pharmacy and Nutrition—similarly to other national authorities—has chosen a restrictive approach (Food and Drug Administration, 2019; Hughes et al., 2022; Hungarian National Institute of Pharmacy and Nutrition, 2022; Woodcock, 2023). In the European Union, foods containing CBD extracts and CBD-enriched foods are considered novel foods, and their distribution is, therefore, prohibited, regardless of their THC content, as cannabinoids are not permitted as novel foods. Only hemp seed and products derived from hemp seed processing (e.g., hemp seed oil) may be used in food supplements, provided that the CBD level does not exceed the impurity limit. Similarly, THC content, regarded as an impurity, needs to be evaluated case by case, taking into account the overall characteristics of the product.

Globally, hemp and CBD products may be subject to varying regulations, which may lead to confusion. According to current European legislation, the generally accepted cannabinoid profile of hemp is 1.5%–3% CBD and less than 0.2% THC. For native and produced hemp seeds, the maximum THC content is 0.3%, while the maximum THC content for hemp seed oil is 0.75% (European Commission, 2023). CBD concentrations can surpass 10% in the United States of America, but THC concentrations cannot exceed 0.3% on a dry-weight basis. In Hungary, products containing less than 10 mg/kg THC are permitted; CBD supplements and novel foods made from cannabis seeds must contain less than 0.2 mg/kg of THC per kilogram of dry weight, and the maximum quantity of CBD is 25 mg/kg (EMCDDA, 2018; Kirilov et al., 2020).

There is public pressure on pharmaceutical authorities to establish clear and concise regulatory frameworks for CBD products, as information regarding product content is frequently restricted or unknown, and lot-to-lot variation and long-term stability are debatable. International organizations involved in the regulation of controlled substances, such as the International Narcotics Control Board (INCB), likewise struggle with the questions and problems associated with these products. Although the INCB has clarified that CBD is not under international control in 2020, the European regulation of *Cannabis* sp. products between Member States is still a matter of debate (Evans, 2020; Adelstone, 2022; Johnson et al., 2022b).

Due to the ongoing issues and debates around CBD oils, it is of public health significance to call the attention of consumers and policymakers to prevent misperceptions about CBD products and reduce the potential harms and health risks associated with their use.

## 2 Aim of this study

Our aim was to analyze the CBD content and label accuracy of CBD oil products available on the internet. Furthermore, we aimed to evaluate the health claims presented on the vendor’s website and on the test-purchased products and discuss the strength of the evidence behind the claimed health benefits.

## 3 Materials and methods

Our study consisted of three sections. First, we conducted an online market analysis, focusing on the content analysis of web stores selling CBD oils for consumers in an effort to identify potentially misleading health claims. Second, selected CBD oils were test-purchased online, and the packaging and labeling of products were evaluated to assess the reliability of the information provided on the delivered products. Finally, product quality was assessed using a quantitative analysis of the CBD content.

### 3.1 Online availability and web shop characteristics

The online availability of CBD oil products was evaluated using a consumer purchase simulation method based on our previously published methodology for risk-based safety mapping of the online pharmaceutical market. This complex risk-based algorithm simulates what consumers can easily find and what websites they are most likely to visit when searching the internet for CBD oils (Vida et al., 2017; 2019; Fittler et al., 2018).

An online search was conducted on Google.hu using the Google Chrome web browser in October 2020. During searches, researchers were not logged into any accounts, and their browsers were configured with default security settings. The national (Hungarian) search term “purchase CBD” (“CBD vásárlás") was used, and the top twenty organic search engine results were recorded. The websites offering CBD oils to consumers were further evaluated. Health claims (“indications”) related to CBD oils and the unit price (EUR/mL) of the products were also recorded. Content available on social media sites was not evaluated in this study. During the search, the authors documented the category of the website (web shop, sites with online information on CBD products, redirect page, social networking site, and others); the language of operation; distributor name; and contact information. Additionally, we evaluated whether a website sold products from a single firm or several brands. The payment choices listed were PayPal, money transfer, and cash on delivery. To estimate the security of an internet connection, the implementation of the Secure Sockets Layer (SSL) protocol was documented, and the safety and dependability of websites were anticipated based on a manual assessment of language and content that emphasized spelling and grammatical problems.

### 3.2 Test purchase

Following a review of the website content, we determined that the majority of websites offered a variety of CBD oils for retail sale. CBD products with the most common concentration (20–50 mg/mL) in the smallest available package size (10 mL) were included to imitate consumers’ purchase decisions. Due to budgetary constraints, 12 products were selected for test purchase. All steps of the purchasing procedure were photographed for further evaluation. The brand name, distributor, purchase identification number, price and shipping cost, method of payment, invoice, and date of arrival were recorded. The inner and outer packaging and any accompanying documentation of the products were photographed upon arrival. The products were stored according to the recommendations on the label or at room temperature in a dry environment, and the oils were tested immediately after opening.

### 3.3 Quantitative analysis and assessment of labeling accuracy

Quantitative analysis was performed using the Shimadzu Nexera X2 UHPLC liquid chromatography system equipped with a vacuum degasser (DGU-20A5R), two binary pumps (LC-30AD), a mixer assembly, an autosampler (SIL-30AC), a column temperature controller (CTO-20AC), and a diode array detector (SPD-M20A). A Kinetex Polar C18 column (100 × 3 mm, 2.6 µm) was used for separation. Run time was 24 min with mobile phase (A) water–methanol 9:1 (v/v) with ammonium formate 50 mM and (B) methanol–water 9:1 (v/v) with ammonium formate 50 mM at a flow rate of 0.5 mL/min. The elution program started with an isocratic step with 75% B; after 19 min, it increased to 100% B in 0.5 min and held for 1 min; and then, it returned to 75% B in 0.5 min and held for the remaining 24:00 min. The temperatures of the autosampler and column oven were 25°C, respectively. The detection was performed at a wavelength of 210 ± 10 nm.

During sample preparation, 300 µL of the product was diluted to 10 mL with methanol and extracted at room temperature using an ultrasonic bath for 10 min. The extracts were diluted 10-fold and subsequently filtrated using a 0.45-µm PTFE syringe filter, and 3 μL was injected into the chromatographic system.

**FIGURE 1:**
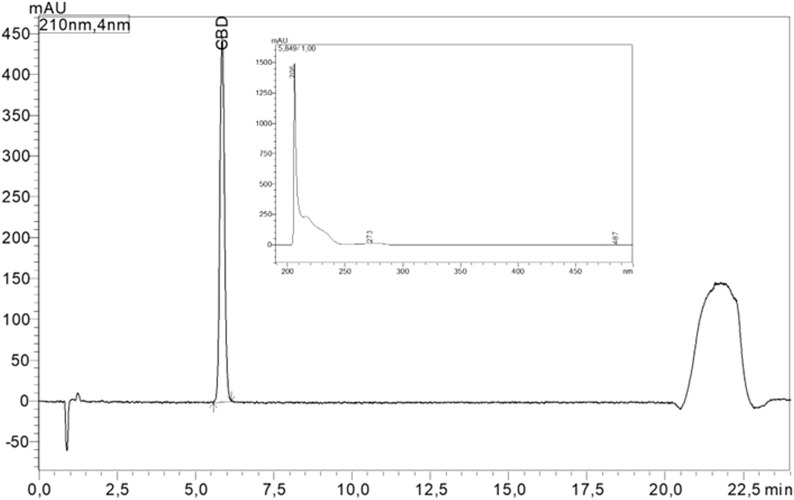
HPLC chromatogram at 210 nm and UV absorbance of CBD.

For CBD quantification, external calibration was applied. Solutions for calibration were prepared with CBD (analytical reference standard, purchased from Cayman Chemical, Michigan, United States; item number 90080, Batch: 0592969-115) in methanol at 1 mg/mL, 0.1 mg/mL, and 0.01 mg/mL. The seven-point calibration curve had an LOD of 29.35 ng/inject and an LOQ of 88.95 ng/inject (Figure 1). The CBD content of the products was calculated and categorized as under-, accurately- and over-labeled with detected CBD concentrations <90%, 90%–110%, and >110% of the labeled value, respectively (Bonn-Miller et al., 2017; Pavlovic et al., 2018; Johnson et al., 2022b; Liebling et al., 2022).

## 4 Results

### 4.1 Online availability and web shop characteristics

From the first twenty search engine results, 18 links were considered relevant, while one informational website about *Cannabis* and one redirecting page, both without purchase options, were excluded from content analysis. After removing duplicates, 16 online stores were identified and included in the analysis. Internet pharmacies were not among the search results (n = 0, 0%). More than half (n/N = 9/16, 56.3%) of online stores offered a wide selection of products, with several companies selling two to seven products on average. Notably, one online store sold 27 distinct types of CBD oils. The remainder (n/N = 7/16, or 43.7%) were limited to a single product or brand. CBD oils with 3%–5% (30–50 mg/mL) concentration were available in all of the online stores included in our analysis, with 10 mL and 30 mL being the most common container sizes. The prices of these products ranged between 15 and 45 EUR; the average unit price for the test purchase products was 1.5–3 EUR/mL; and the total price, including shipping, was between 23.7 and 47.7 EUR (see Table 2). Two products were unregistered at the National Institute of Pharmacy and Nutrition (“BioBloom CBD 400 mg (4%) organic Hemp drops” and “Biofora Harmony premium 5% (500 mg) CBD oil with Hemp oil”). Through additional online research, we determined that 12 products were available from web shops outside of Hungary and are, therefore, regarded as worldwide available, whereas just four products were offered only in Hungary.

Hundred percent (n/N = 16/16) of the web shops had an SSL certificate, providing an encrypted connection between the parties, and 81.2% (n/N = 13/16) had a privacy policy statement about data management. In 12 online stores, we were able to pay via PayPal, wire transfer, or cash on delivery, whereas three accepted only cash on delivery. The language of the web stores was Hungarian; however, two (n/N = 2/16, 12.5%) websites contained improper Hungarian language material with grammatical mistakes. One of these (www.cbdcibdol.hu) was a 15-language multilingual website whose poor-quality text may have been the result of an automated translation tool. We found 10 (n/N = 10/16, 62.5%) web stores with Hungarian addresses, three (n/N = 3/16, 18.8%) with international addresses (one Dutch and two Czech), and three (n/N = 3/16, 18.8%) with no physical location indicated and only a telephone number and email address.

### 4.2 Website content analysis of product information and health claims

Critical evaluation of health claims—including potential benefits and adverse effects—is of utmost importance, as potential consumer harm and adverse effects may be associated with the improper application of these products or the application for health conditions without medical supervision, in addition to or *in lieu* of medicinal therapy. Consequently, the information provided to consumers during the purchasing process is a vital element of consumer safety. As these products were on the market as food supplements in the year of purchase, national and international rules prohibit attributing any therapeutic benefit to these products (Kušar et al., 2021). Furthermore, CBD has no acknowledged or pending health claims (European Parliament, 2006).

Upon assessment of the 16 web shops, three (n/N = 3/16, 18.8%) contained no reference to the potential therapeutic application of CBD oils, two (n/N = 2/16, 12.5%) listed health claims within the product information page, while the majority (n/N = 10/16, 62.5%) displayed potential health-related benefits indirectly on their website, separately from the product purchase page. In the case of one online store (n/N = 1/16, 6.3%), we identified a COVID-19-related application. The number of health claims associated with CBD oils that were highlighted directly on the page of the product ranged from 0 to 13 (mean = 1.9 ± 4.5), and when additional pages of the website were analyzed, the range of health claims was between 0 and 56 (mean = 15.4 ± 16.3).

We assessed health claim information available in online stores by searching for medical conditions and then ranked these claims based on their frequency. As shown in Table 1, we have identified a total of 30 categories of purported “indications” for the purchased food supplements. Additional indications, such as epilepsy, which is an authorized indication for a cannabidiol-based medicine, were also commonly mentioned, possibly as a direct-to-consumer marketing technique to raise demand for these products among certain patient groups.

**TABLE 1:** Occurrence of direct or indirect health claims and indications on 16 web shops offering CBD oils for sale.

Health claims categorized according to the medical conditions	Occurrence (%)
Autism, anxiety, depression, panic attacks, and bipolar disorder; cardiovascular related issues: heart condition, blood pressure, cholesterol, and arrhythmia (*n* = 2)	56.25
Regulation of the CNS; regulation of circadian clock; epilepsy; dependences; anti-inflammatory effects; symptomatic treatment in case of cancer; digestion; inflammatory bowel disorders (*n* = 6)	50.00
Regulation of nociception; regulation of the endocrine system (diabetes and obesity) (*n* = 2)	43.75
Against neurodegenerative diseases, e.g., Alzheimer’s disease, Parkinson’s disease, dementia, and sclerosis; regulation of immune function; treatment of allergies; increase in the amount of muscle tissue; regulation of the reproductive system (e.g., PMS) (*n* = 4)	37.50
Neuroprotective effect and positive effect in case of CNS injury (trauma and stroke); rheumatology treatment (*n* = 2)	31.25
Positive effects on memory, learning, and mood; prevention and treatment of osteoporosis (*n* = 2)	25.00
Regulation of motoric functions in Huntington’s disease; regulation of aggression; regulation of appetite and body weight (increase); antiemetic effect; against migraine; regulation of the endocrine system; regulation of liver function; in dermatology (psoriasis and acne) (*n* = 8)	18.75
Regulation of body temperature; AIDS symptoms; gout (*n* = 3)	12.50
Glaucoma (*n* = 1)	6.25

### 4.3 Sample acquisition and delivery

In December 2020, 12 CBD oil products identified by a previous market analysis were obtained from online retailers for testing purposes. The majority of products (66.5%, n/N = 8/12) came within 1 day, while the remainder arrived within 7 days. All test purchases were attached to an invoice; seven electronic, three paper-based and, in the case of two products, in both formats. The majority of the products, 75%, were shipped from Hungary (n/N = 9/12), and one product was shipped from Germany. Shipping information was unavailable for two products (16.6%, n/N = 9/12). Only four products (33.3%, n/N = 4/12) had a product information leaflet, and only one product (8.3%, n/N = 1/12) included application-specific information.

Upon the assessment of the packaging, we observed that in the case of nine products (n/N = 9/12, 75.0%), the national authority notification number was not highlighted, and instructions in the national language of the country of delivery (Hungarian) were not available for three CBD oils (n/N = 3/12, 25%). No instructions were supplied for the remaining three products (n/N = 3/12, 25%), which raises the risk of inappropriate application and dosing. The collected information regarding the CBD oils purchased for testing is shown in Table 2.

**TABLE 2:** Main characteristics of CBD products purchased from selected web shops.

ID#	Purchase ID	Product name	Distributor	Total price and payment method	Shipping time	Instructions for use	Shipping country and address
1	3121389578	CBD oil 1,000 mg, 30 mL “high dose”	United States medical	47.7 USD	1 day	National language + warnings	United States Medical Kft. 2040, Budaörs, Puskás Tivadar út 10 (Hungary)
				PayPal			
2	#41969	ENECTA 3% wide-spectrum CBD oil 10 mL	Enecta	23.7 on arrival	1 day	National language + warnings	CBDrendeles-Fullfilled by Webshippy East Gate Business Park C/2 2,151 Fót (Hungary)
3	B00375958	Cibdol 5% CBD oil	Cibdol	29.9 USD	6 days	National language + warnings	Portpayé 60544 Frankfurt Allemagne Send back address: 36243 Niederaula (Germany)
				PayPal			
4	21119489	LOVE HEMP^®^ 600 MG CBD oil drops, 30 mL wild cherry	Love Hemp	38.0 USD on arrival	1 day	National language + warnings	Hempstore Kft. Nagytarcsa Vadrózsa utca 5. 2,142 (Hungary)
5	CBD-00000794	BioBloom 10 mL 400 mg Organic Hemp oil 10 mL	Biobloom	36.7 USD	4 days	Not available	Unidentifiable
				PayPal			
6	TNW502885	Honey Heaven CBD Oil 500 mg CBD (10 mL) 5%	Honey Heaven	30.8 USD on arrival	1 day	National language	Zox trade Kft 1 Szent Márton u. 13. 9,700 Szombathely (Hungary)
7	20603911	Candorra CBD Hemp oil 5%, 10 mL	Cannadorra	33.6 USD	7 days	English language + warnings	Frogman s.r.o. Budaörs Gervay Mihály utca 9–11 (Hungary)
				PayPal			
8	#10455	CbdBase Hemp complex CBDA/CBD Oil, 5%, 10 mL 500 mg	Cbdbase	36.3 USD	1 day	English language + warnings	Unidentifiable
				PayPal			
9	#1618	CBD Oil 10 mL/500 mg SATIQUM	SATIQUM	28.5 USD	6 days	English language + warnings	Gnath Hunt Kft. 1,152 Budapest Szentmihályi út 167–169 (Hungary)
				PayPal			
10	#4390	Medijuana Ultrasoft FULL Spectrum CBD oil, 5% (10 mL)	Medijuana	29.5 USD	1 day	Not available	Winning Trade Kft. Szeged Pásztor u. 13. 6,725 (Hungary)
				PayPal			
11	#12812	ENDOCA 300 mg CBD Hemp oil (3%) “heated”	Endoca	26.1 USD	1 day	Not available	Hempstar-Fullfilled by Webshippy East Gate Business Park C/2 2,151 Fót (Hungary)
				PayPal			
12	1333	BIOFORA HARMONY Premium 5% (500 MG) CBD OIL with Hemp oil 10 ML	Biofora Harmony	29.7 USD	1 day	National language + warnings	Biofora Herbal kft. Budapest Liszt Ferenc tér 10. 1,061 (Hungary)
				PayPal			

### 4.4 Quantitative analysis and assessment of labeling accuracy

**FIGURE 2:**
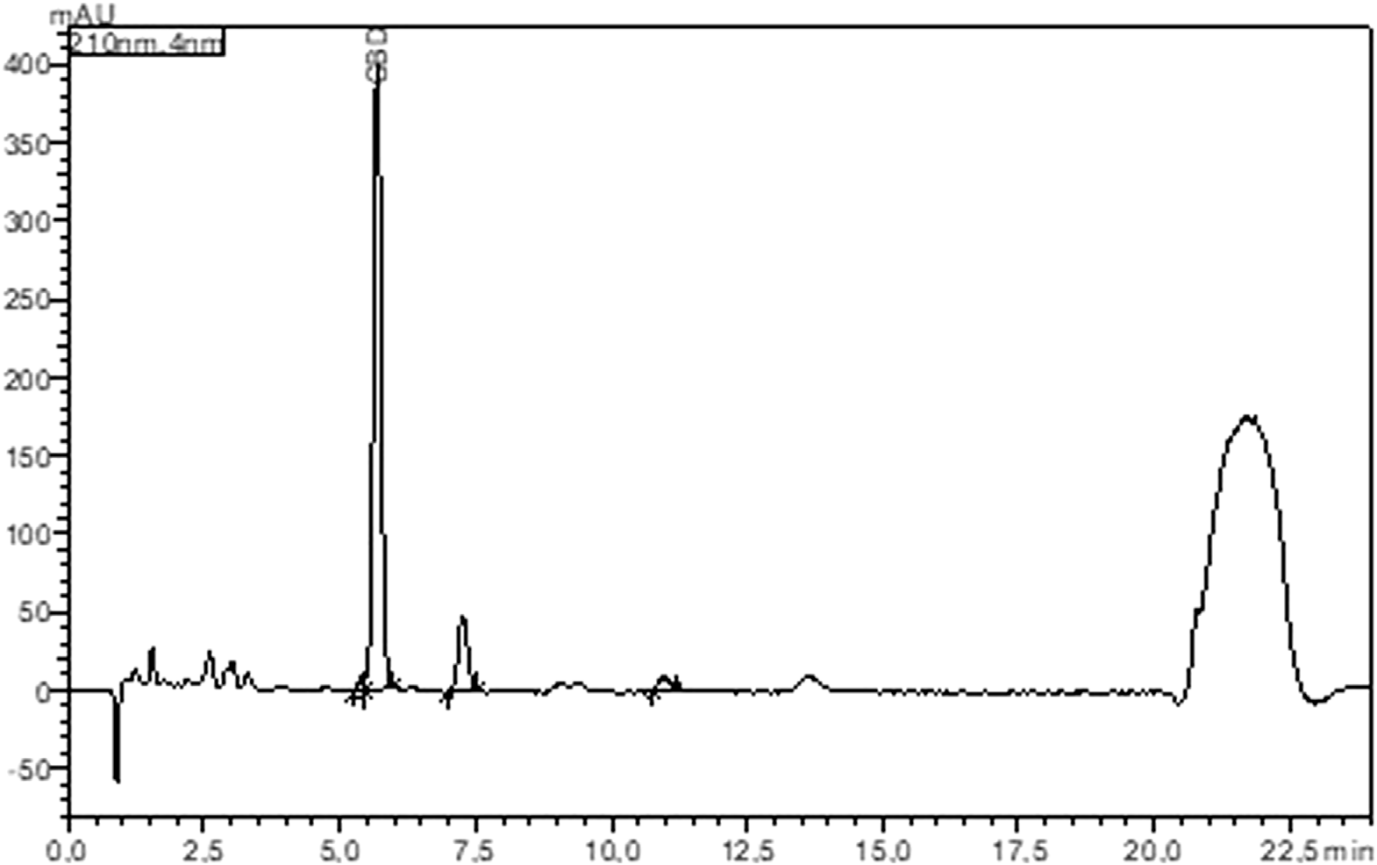
HPLC chromatogram at 210 nm of ID#11 product (ENDOCA 300 mg CBD hemp oil (3%) “heated”).

CBD concentration ranged from 19.58 mg/mL to 54.09 mg/mL across the twelve items that were purchased and evaluated (Figure 2). The concentrations indicated on the containers ranged from 20 mg/mL to 50 mg/mL. Based on a quantitative examination of CBD concentration, 8.33% (1/12) of the items were under-labeled, while 41.67% (5/12) were over-labeled. Of the products evaluated, 50% were appropriately labeled (within 10%). The under-labeled product (LOVE HEMP^®^ 600 MG CBD oil drops—30 ML wild cherry) contained 113.80% CBD, which was greater than the labeled concentration (20 mg/mL against 22.76 mg/mL). With a concentration of 19.58 mg/mL, CbdBase Hemp Complex CBDA/CBD Oil 5% - 10 mL 500 mg had the lowest concentration (39.16%). The labeling accuracy of CBD oils purchased for testing is shown in Table 3.

**TABLE 3:** Labeling accuracy and number of health claims of CBD oils test-purchased over the internet.

ID#	Product name	Label claim mg CBD/mL	Observed mg CBD/mL ± SED (mg/mL)	Difference mg CBD/mL	Percent of label claim (%)	Category	Number of health claims	
							Directly or indirectly on the seller website	On the delivered product
1	CBD oil 1,000 mg–30 mL “high dose”	33	31.67 ± 2.34	−1.33	95.97	Accurately labeled	16	None
2	ENECTA 3% wide-spectrum CBD oil 10 mL	30	29.58 ± 0.23	−0.42	98.60	Accurately labeled	37	None
3	Cibdol 5% CBD oil	50	54.09 ± 0.14	+4.09	108.18	Accurately labeled	None	None
4	LOVE HEMP^®^ 600 MG CBD oil drops—30 ML wild cherry	20	22.76 ± 0.24	+2.76	113.80	Under-labeled	None	None
5	BioBloom 10 mL 400 mg Organic Hemp oil 10 mL	40	24.71 ± 1.18	−15.29	61.78	Over-labeled	18	None
6	Honey Heaven CBD Oil 500 mg CBD (10 mL) 5%	50	43.18 ± 0.70	−6.82	86.36	Over-labeled	13	None
7	Candorra CBD Hemp oil 5% 10 mL	50	50.10 ± 0.91	+0.10	100.20	Accurately labeled	15	None
8	CbdBase Hemp complex CBDA/CBD Oil—5% - 10 mL 500 mg	50	19.58 ± 0.35	−30.42	39.15	Over-labeled	7	None
9	CBD Oil 10 mL/500 mg SATIQUM	50	26.62 ± 1.00	−23.38	53.24	Over-labeled	7	None
10	MEDIJUANA ULTRASOFT FULL Spectrum CBD oil—5% (10 mL)	50	50.51 ± 3.85	+0.51	101.02	Accurately labeled	13	None
11	ENDOCA 300 mg CBD Hemp oil (3%) “heated”	30	25.92 ± 1.16	−4.08	86.40	Over-labeled	20	None
12	BIOFORA HARMONY Premium 5% (500 MG) CBD OIL with Hemp oil 10 ML	50	47.42 ± 1.99	−2.58	94.84	Accurately labeled	28	None

## 5 Discussion

In 2015 and 2016, the FDA conducted the first research assessing the content and labeling accuracy of CBD products and issued warning letters (warning of products with negligible or less than 1% CBD content) (Food and Drug Administration, 2021). In 2016, Bonn-Miller et al. ordered 84 CBD products and analyzed their content and labeling. Only 31% of the products were within ±10% of the stated CBD content (concentration ranged between 0.10 mg/mL and 655.27 mg/mL), and less than 50% (n = 18) were appropriately labeled. In general, the concentration of unlabeled cannabinoids was modest; nonetheless, THC was detected in 21.43% (18/84) of the products purchased (up to 6.43 mg/mL) (Bonn-Miller et al., 2017).

The officers of the Mississippi Bureau of Narcotics in 2020 purchased CBD-containing products from offline retailers in the state of Mississippi, according to a separate study. Of the 25 oils and electronic cigarette vaping products analyzed, 88% (n = 22) contained measurable quantities of CBD, and only three products (12%) were within 20% of the label claim. Three products contained THC, and four products (three vaping liquids) were adulterated with synthetic cannabinoids (4-fluoro MDMB-BUTINACA, 5-fluoro MDMB-PICA, and 5-fluoro MDMB-PINACA) (Gurley et al., 2020). Topical CBD products were purchased in local shops and online in 2020 in Baltimore, Maryland, and of the 105 products, 8% were over-labeled, 58% were under-labeled, and 24% were accurately labeled for CBD (applying the 10% rule like the other publications). THC content was detected in 35% of the products (less than 0.3%). The authors highlighted the misleading therapeutic or cosmetic claims (Spindle et al., 2022).

However, these findings are not country-specific and reflect a worldwide trend since other European studies have shown the same discrepancies in labeling accuracy as the International Cannabis and Cannabinoids Institute reported in 2017 (Cannabis and Cannabinoids Institute, 2017). In a Dutch study, 46 cannabis oil products (29 home-made and 17 web-purchased) were collected directly from consumers, and it was found that only 46% of the products had label information (CBD/THC content), seven products did not contain any measurable phytocannabinoids, and 26 samples (57%) had a THC content >1%, with one sample reaching 57.5% (575 mg/mL), whereas the CBD content ranged from 0.1% to 27% (1 mg/mL and 270 mg/mL) (Hazekamp and Epifanova, 2017). In 2018, an Italian study assessing the overall quality of CBD oil products purchased online found that only five of 14 products contained at least 10% of the CBD amount stated on the label. The CBD content varied between 0.24 and 4.96 w/w%. Additional investigation indicated that 12 of 14 contained THC (Pavlovic et al., 2018). In a 2019 study from the UK, Liebling et al. analyzed 29 products purchased online and offline from 27 different suppliers to test cannabinoid content, heavy metals, and residual solvents. Regarding the CBD content, only 11/29 (38%) products were within 10% of the labeled content. Ten percent of the overall cannabinoid content was comprised of non-CBD phytocannabinoids, including THC (0%–0.22%), THCA, THCV, CBN (0%–0.12%), CBG, CBGA, and CBC, when the other cannabinoid components were evaluated, including THC (0%–0.22%), THCA, THCV, CBN (0%–0.12%), CBG, CBGA, and CBC. Some products contained residual solvents like n-pentane, ethanol, ethyl acetate, isopropanol, heptane, and cyclohexane; in addition, small quantities of lead (0.01–0.24 ppm) and arsenic (0.01–0.06 ppm) were found in some products. All values were below the ICH guidelines for medicinal products but above safety levels of the food limit (Liebling et al., 2022). In 2021, a Belgian study investigated 18 CBD oils that were seized by inspectors and concluded that 45% of the analyzed items met the acceptance limits for the CBD content. The provenance of the products ranged from the Netherlands to Switzerland to Barcelona. Δ^9^-THC was not detected in the products analyzed (Duchateau et al., 2021).

Another study in the United States investigated hemp-derived products from both online and local retailers in Kentucky (n = 80) from 2 April to 9 May 2021. Unique to the experiment were the use of authorized CBD medicinal products as a positive control (Epidiolex) and the inclusion of only oil products. Of the 80 products, 31% were under-labeled, 15% were over-labeled, and 54% were accurately labeled. The CBD concentration varied between 9.3 and 60.5 mg/mL (24.1 ± 15.3 mg/mL). The THC levels were presented in another publication, where the authors reported that the Δ^9^-THC concentration of the 51 analyzed products (64%) ranged from 0.008 mg/mL to 2.071 mg/mL (Johnson et al., 2022a; Johnson et al., 2022b). In a study led by a pharmacy student in the southwest Wisconsin area in 2021, 39 cannabidiol products were purchased from local retail shops, and of the 11 oils, only 36.36% (n = 4) were appropriately labeled. THC was detected in 54.55% (n = 6) of the oils with a maximum concentration of 0.2% w/v (Miller et al., 2022). As part of an international study conducted in 2020–2021, 24 samples were purchased online from Austria, France, Germany, Great Britain, Luxembourg, the Netherlands, and Spain. Seven samples were within 10% of the reported CBD values (CBD and CBDA), sixteen samples were over-labeled, and one sample had a concentration that was more than five times lower than the declared concentration. One sample had a THC concentration above 0.2% w/w (the legal limit in most European countries) (Schneider, 2021).

During our literature search, we also discovered a review of label accuracy studies in which, based on the results of five studies, it was concluded that the proportion of correctly labeled products ranged from 17% to 86% and that further well-designed research is required in this field due to the paucity and heterogeneity of the available studies. In this product category, reporting the potential for adverse health effects and practicing pharmacovigilance are crucial (Bonn-Miller et al., 2017; Stevenson, 2018; Blebea et al., 2019; Herbst and Musgrave, 2020; Oldfield et al., 2021; Vandrey et al., 2022).

The ratio of accurately labeled products in our study sample was higher (*n* = 6; 50%) than in the previously published literature from the United States (31% by Bonn-Miller et al. in 2016, 36.36% by Miller et al. in 2021, and 24% by Spindle et al. in 2022) or Europe (23.5% by Hazekamp and Epifanova in 2017 in the Netherlands, 35.7% by Pavlovic et al. in 2018 in Italy, 38% by Liebling et al. in 2019 in the United Kingdom, 29.2% by Schneider in 2021, and 48% by Duchateau et al. in 2021 in Belgium). Similar results were found and published by Johnson et al. with 54% labeling accuracy in 2022 in the United States. (Bonn-Miller et al., 2017; Hazekamp and Epifanova, 2017; Pavlovic et al., 2018; Duchateau et al., 2021; Schneider, 2021; Johnson et al., 2022a; Johnson et al., 2022b; Liebling et al., 2022; Miller et al., 2022; Spindle et al., 2022).

Chronic pain and spasticity, multiple sclerosis, treatment-resistant epilepsy, nausea and vomiting due to chemotherapy, weight gain in HIV infection, sleep disorders, Tourette syndrome, anxiety, post-traumatic stress disorder, and schizophrenia are among the approved indications or applications of authorized CBD medicinal products. (Whiting et al., 2015; EMCDDA, 2018; Freeman et al., 2019; VanDolah et al., 2019; Sholler et al., 2020).

Although some cannabinoids have beneficial effects, CBD products that are not medications are prohibited from using therapeutic indications and health claims in their marketing. Similar to our findings, other studies have indicated that regulation violations occur often on the food supplement markets in the United States and Europe (Evans, 2020; Zenone et al., 2021; Amann et al., 2022; Bilbao and Spanagel, 2022; Spindle et al., 2022). A good and comparable example is anxiety, which, in our study, emerged as a leading “indication” for CBD oils. Similarly, a study by Soleymanpour et al. (2021) analyzed Twitter medical claims related to CBD-containing products and found pain, anxiety disorders, sleep disorders, and stress to be the four main “therapeutic” applications claimed on the social media platform (Soleymanpour et al., 2021).

CBD oils usually have a lower concentration of CBD than medicinal products in clinical trials; therefore, claims on efficacy should be handled with precautions. When evaluating the potency of CBD oils and their potential for health risk, a dose regimen and content analysis comparable to ours, as well as a comparison with approved medications containing the same substance, might be helpful (Table 4).

**TABLE 4:** Suggested dosing and maximum CBD content of the ordered products compared to authorized medicine.

Product name	Suggested dosing from the manufacturer	Observed mg CBD/mL (approximately 20 drops)	Maximum CBD content in one product (mg)	Maximum daily dose in the authorized medicines
CBD oil 1,000 mg–30 mL “high dose”	5–200 mg/day	31.67 ± 2.34	950.1 ± 70.2	Epidiolex: 20 mg/kg/day; Sativex: 30 mg/day for CBD
ENECTA 3% wide-spectrum CBD oil 10 mL	5 mg/day	29.58 ± 0.23	295.8 ± 2.3	
Cibdol 5% CBD oil 10 mL	32.454 mg/day	54.09 ± 0.14	540.9 ± 1.4	
LOVE HEMP^®^ 600 MG CBD oil drops—30 ML wild cherry	10–70 mg/day	22.76 ± 0.24	682.8 ± 7.2	
BioBloom 10 mL 400 mg Organic Hemp oil 10 mL	-	24.71 ± 1.18	247.1 ± 11.8	
Honey Heaven CBD Oil 500 mg CBD (10 mL) 5%	27–200 mg/day	43.18 ± 0.70	431.8 ± 7.0	
Candorra CBD Hemp oil 5% 10 mL	15–37.5 mg/day	50.10 ± 0.91	501.0 ± 9.1	
CbdBase Hemp complex CBDA/CBD Oil—5% - 10 mL 500 mg	2–30 mg/day	19.58 ± 0.35	195.8 ± 3.5	
CBD Oil 10 mL/500 mg SATIQUM	9–27 mg/day	26.62 ± 1.00	266.2 ± 10.0	
MEDIJUANA ULTRASOFT FULL Spectrum CBD oil—5% (10 mL)	-	50.51 ± 3.85	505.1 ± 38.5	
ENDOCA 300 mg CBD Hemp oil (3%) “heated” 10 mL	-	25.92 ± 1.16	259.2 ± 11.6	
BIOFORA HARMONY Premium 5% (500 MG) CBD OIL with Hemp oil 10 ML	4–14 mg/day	47.42 ± 1.99	474.2 ± 19.9	

Admittedly, we could not identify toxic levels of the CBD content or recommended dose, and the preclinical and clinical data from medicine trials cannot be generally applied to other health products. However, the uncontrolled product quality in this market and unsupervised application or combination with other *cannabis* or addictive substances (e.g., alcohol and illegal substances) pose definite health risks. Uncontrolled consumption (especially when the product lacks application and dosage information) of a new ingredient regulated as a drug for which there is no EFSA novel food recommendation should not be promoted and regarded as a safe phenomenon. From a legal perspective, these products are regarded as counterfeit medicines: food supplements containing active pharmaceutical ingredients (Freeman et al., 2019; Bansal et al., 2020; Nasrin et al., 2021; Johnson et al., 2022b).

The EFSA NDA panel recently defined a 4.3 mg/kg bw/day lowest observed adverse effect level (LOAEL) value for cannabidiol in humans, and the authors suggest that CBD products available in the EU market as food supplements or other non-medical health products should be considered unsafe. Risk assessment should be implemented with the guidance of two values: a health-based guidance value (HBGV) of 10 mg/day, and products exceeding this should be titled “unfit for consumption,” while products exceeding human LOAEL should be considered “injurious to health.” When applying these limits (see Table 4) to our study samples, all of our ordered products can be considered as “unfit for consumption” as their recommended dosing exceeds the 10 mg/day limit (Lachenmeier et al., 2023).

The use of CBD products should be advised with caution for several patient or consumer groups, including young adults (18–25 years old) due to the unknown effects of CBD on developing brains; people with coexisting psychiatric conditions; elderly people taking multiple medications, as CBD may increase the risk of falls; people with decreased liver function or liver disease; women who are pregnant or nursing; and people who have allergies to cannabis or components in CBD products (Health Canada, 2022).

Given that CBD products are probably used under unsupervised circumstances, it is imperative to provide accurate product information about how to use and dose these items. Nevertheless, our study also demonstrated that consumers are more likely to take the wrong dosage, which is consistent with prior label accuracy studies from the US, Europe, or other nations. Therefore, these products can have serious negative effects on health when used by sensitive patient groups (such as those who have epilepsy) (Health Canada, 2022).

The Canadian government appointed a scientific committee to examine cannabis-containing goods, and the board made the following recommendations: for healthy adults, oral administration of CBD at doses ranging from 20 mg per day (mg/day) to a maximum of 200 mg/day is safe and tolerable for short-term use (a maximum of 30 days; e.g., enzyme-induction-related adverse effects require prolonged exposure, such as greater than 21 days), as long as they discuss the use of all other medications and substances with their pharmacist (Health Canada, 2022). It is important to take note of regional variations because the Australian standard from 2020 identified a low dose as 1 mg/kg/day or roughly 60 mg/day. It is also recommended that companies include warnings regarding special patient groups like pregnant and breastfeeding women, allergies, patients taking multiple prescribed medications, and the potential for drug interactions. Additionally, it is advised to include in the packaging of health products containing CBD clear dosing instructions and warnings of potential side effects, as they are dose-dependent. The committee recommends that since there are no conclusive studies that have validated its use for such indications, health products containing cannabis should bear a warning stating that they are not designed to help reduce consumption of opioids or alcohol. Along with the regulatory overview and change, the education of the public through awareness campaigns should also be carried out (Australian Government Department of Health and Aged Care Therapeutic Goods Administration, 2020). Labels on these products should encourage consumers to report adverse reactions on a defined platform or to the pharmacist. Finally, it is advisable to restrict the availability of these products; for example, health products containing CBD should only be available in pharmacies (National Mental Health and Substance Use Policy Laboratory, 2023). These recommendations might also be helpful to other countries that are having trouble regulating CBD products.

Since there was no significant history of consumption in the EU prior to May 1997, CBD and other cannabinoids were confirmed to fall under the novel food legislation in 2019. As a result, CBD oils must first undergo evaluation and authorization before they can be utilized as an ingredient in food or dietary supplements. The data currently available are insufficient to determine the definite no observed adverse effect level (NOAEL) and the LOAEL values, two crucial metrics for evaluating the toxicity of CBD. Furthermore, while evaluating CBD as a novel food, interactions should be taken into account due to the intricacy and importance of CBD receptors and pathways. Not surprisingly, the EFSA Panel could not conclude the safety of CBD as a novel food in 2022. In 2021, the Committee on Toxicity of Chemicals in Food, Consumer Products, and the Environment (COT) suggested setting a daily maximum for food exposure to CBD of 1 mg/kg body weight (The Committee on Toxicity of Chemicals in Food, 2021; EFSA Panel on Nutrition et al., 2022). Although there is a general increase in awareness and scrutiny from a regulatory perspective, distributors and companies have found other ways to sell the products, such as cosmetics. Our research group found a CBD product with a Cosmetic Products Notification Portal (CPNP) notification on their website (see United States MEDICAL CBD). Refer to the current legislation on hemp products and their CBD and THC content provided in Table 5.

**TABLE 5:** Current legislation of hemp products and their CBD and THC content globally (Drug Enforcement Administration, 2018; EMCDDA, 2018; Australian Government Department of Health and Aged Care Therapeutic Goods Administration, 2020; Dabrowska et al., 2020; Kirilov et al., 2020; Health Canada, 2022; European Commission, 2023; National Mental Health and Substance Use Policy Laboratory, 2023).

Region	Regulation on the CBD content	Regulation on the THC content
United States, Canada	Hemp derivatives can have high concentration of CBD (maybe more than 10%); however, foods and dietary supplements cannot contain CBD!	The Farm Bill defined hemp as *Cannabis sativa* L. with delta-9 THC concentration not more than 0.3% (on a dry-weight basis)
EU	All products, made from industrial hemp and CBD-containing products are legal. Foods and dietary supplements cannot contain CBD!	Less than 0.2% THC
Hungary	All products, made from industrial hemp and CBD-containing products are legal (maximum 25 mg/kg). Foods and dietary supplements cannot contain CBD!	Less than 0.2% THC
Australia	Hemp seed products that contain less than 0.01% CBD are allowed to be sold directly without a prescription	All hemp products must contain less than 0.005% THC for it to be considered legal

### Strengths and limitations

Although numerous studies have evaluated CBD products sold over the internet in previous years, this is the first Central European study. Additionally, we focused on the evaluation of health claims while assessing the correctness of the labeling on the purchased products. Last, the purpose of this study is to provide an up-to-date, complete overview and discussion of the international relevance of marketing and regulatory concerns pertaining to CBD oils. Admittedly, there are a few limitations to our study that we must acknowledge. First, we only considered oils and excluded all other CBD products; however, at the time of purchase, oils were the most popular and readily available. Second, reporting additional cannabinoids would have provided more comprehensive results.

## 6 Conclusion

In recent years, CBD products have gained global attention due to their inconsistent labeling and unregulated marketing, which pose a significant threat to consumer and patient safety since their diverse compositions might result in toxicities and drug interactions. The likelihood of serious adverse effects, such as liver failure, should highlight the dangers associated with the use of illegally marketed CBD oils. The improvement of pharmacovigilance, such as customers reporting adverse reactions to non-medicinal items (including dietary supplements), can be a valuable method for reducing risks. To build the regulatory framework for CBD containing products, additional research, including phytochemical investigations using validated methodologies, clinical and real world safety studies, is required.

## Data Availability

The original contributions presented in the study are included in the article/Supplementary Material; further inquiries can be directed to the corresponding author.
