# Supplementary material for: Safety and risks of CBD oils purchased online: unveiling uncertain quality and vague health claims
Source: Front Pharmacol. 2023 Dec 14;14:1273540. doi: 10.3389/fphar.2023.1273540 (PMC10773908; doi:10.3389/fphar.2023.1273540)
Supplement: Supplementary file 1 [file DataSheet1.PDF]

Supplementary material

UHPLC chromatograms of Investigated CBD products

#1 – CBD oil 1000 mg – 30 ml „high dose"

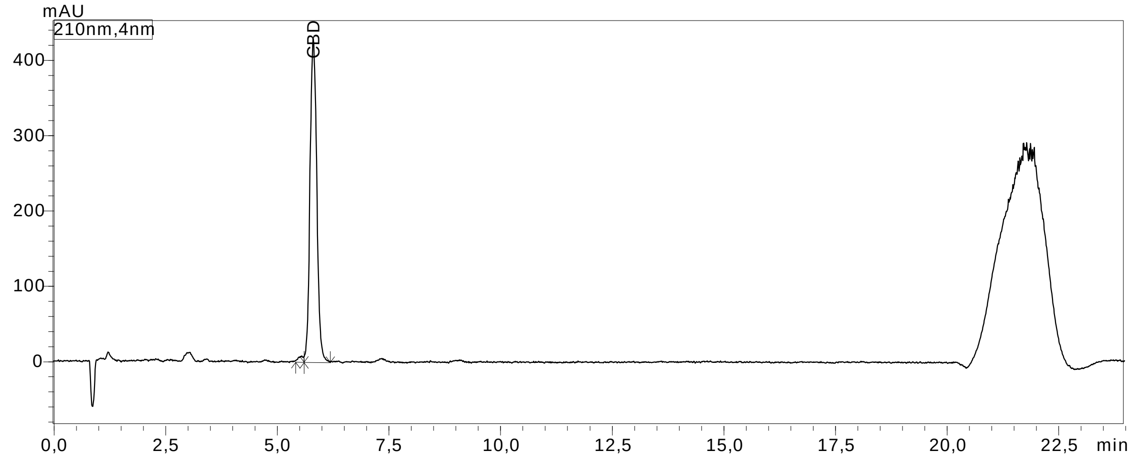

#2 – ENECTA 3% wide spectrum CBD oil 10 ml

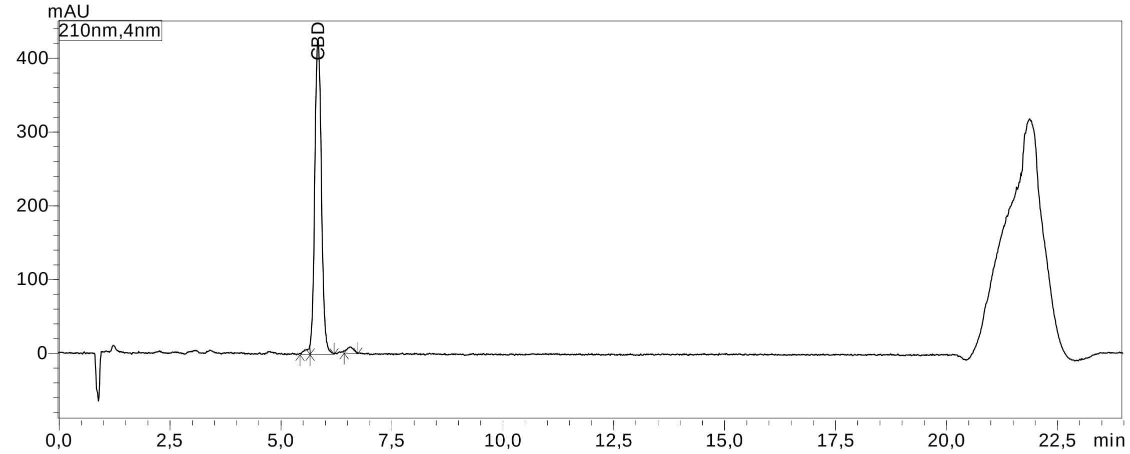

#3 – Cibdol 5% CBD oil

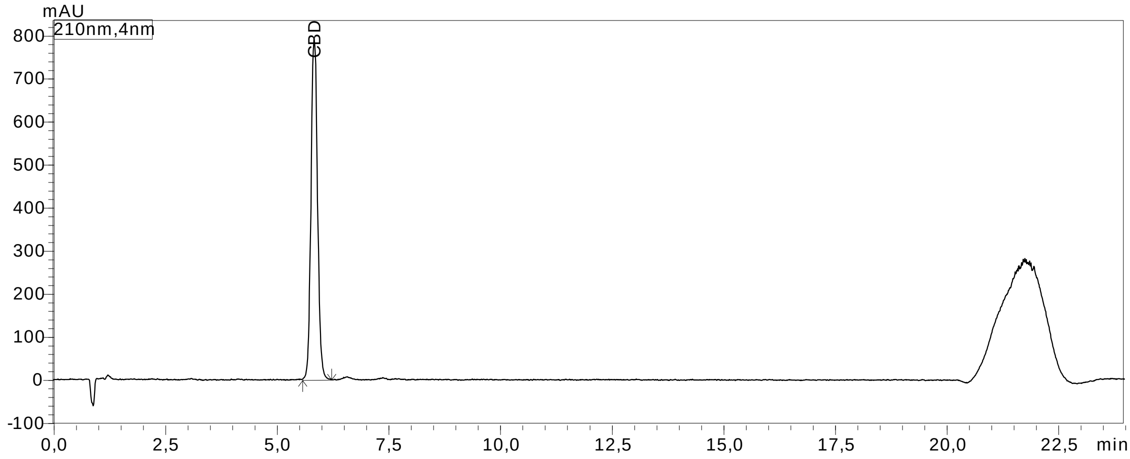

#4 – LOVE HEMP 600MG CBD oil drops – 30 ML wild cherry

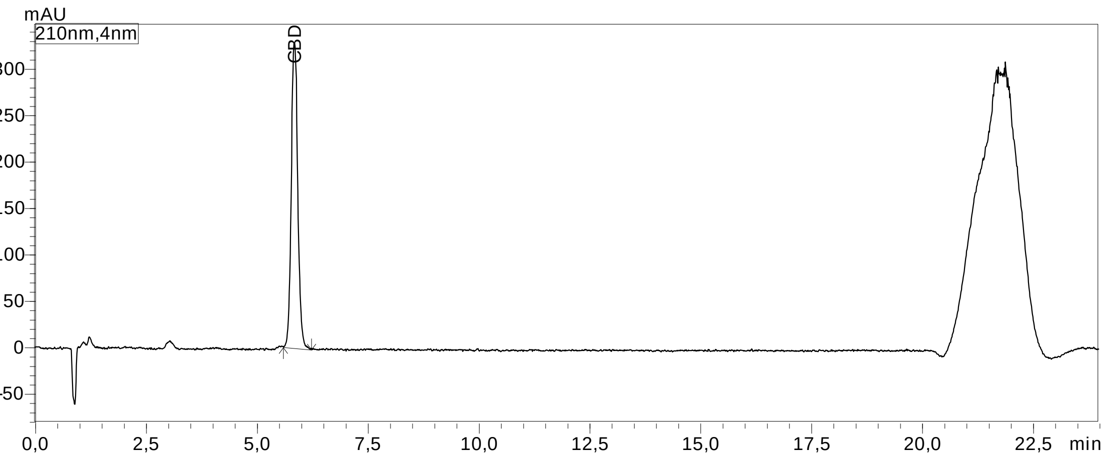

#5 – BioBloom 10 ml 400 mg Organic Hemp oil 10 ml

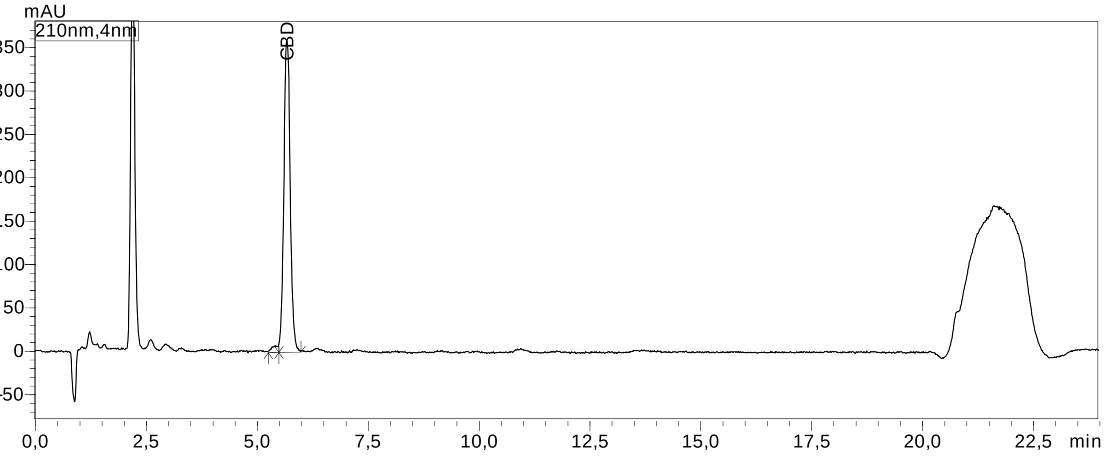

#6 – Honey Heaven CBD Oil 500 mg CBD (10 ml) 5%

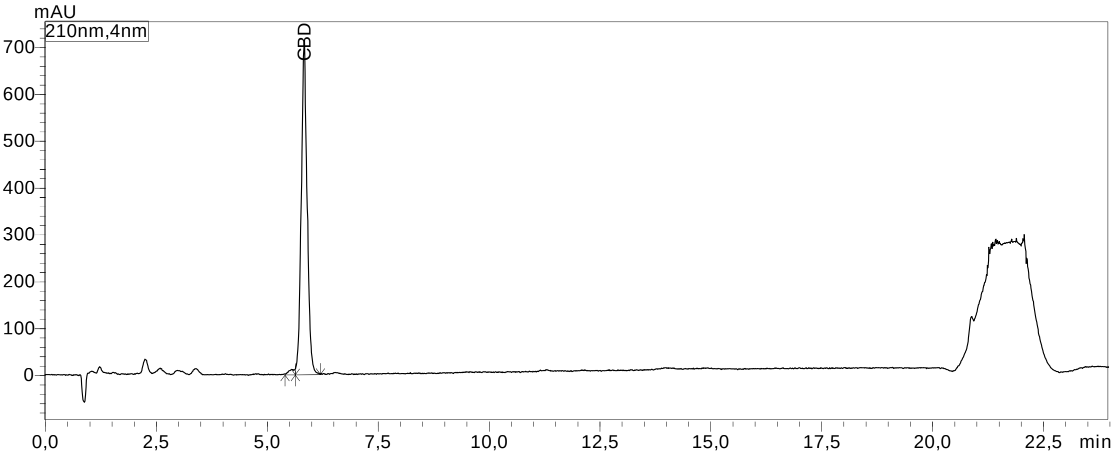

#7 - Candorra CBD Hemp oil 5% 10ml

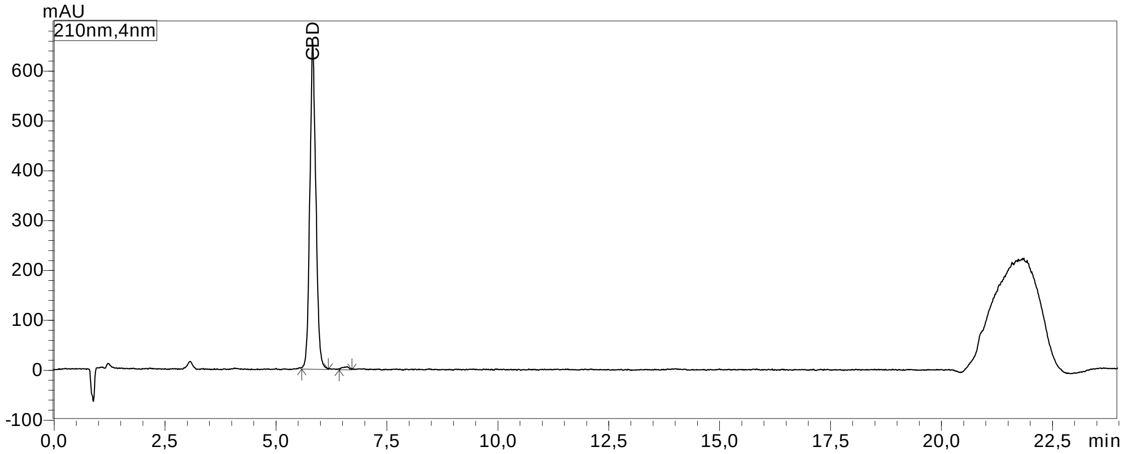

#8 – CbdBase Hemp complex CBDA / CBD Oil – 5% - 10ml 500 mg

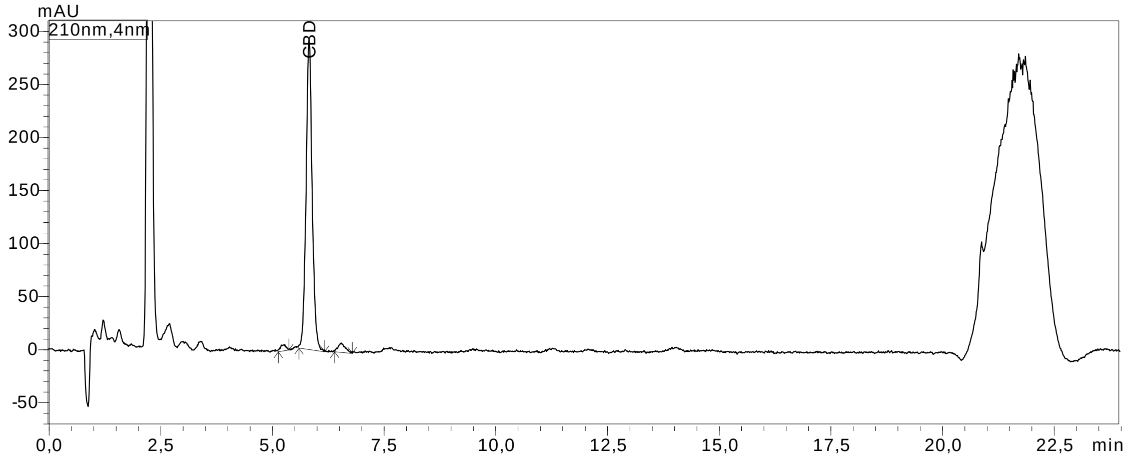

#9 – CBD Oil 10 ml/500 mg SATIQUM

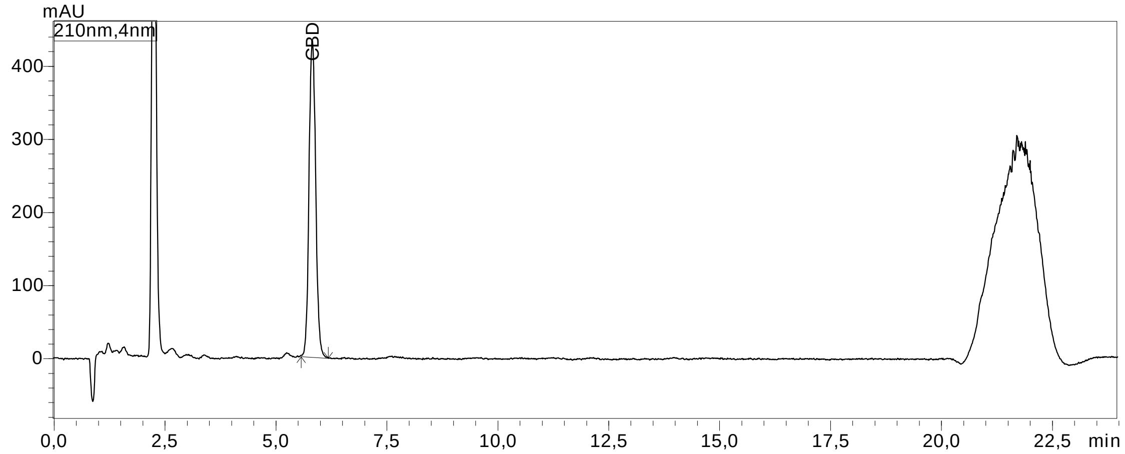

#10 – MEDIJUANA ULTRASOFT FULL Spectrum CBD oil – 5% (10 ml)

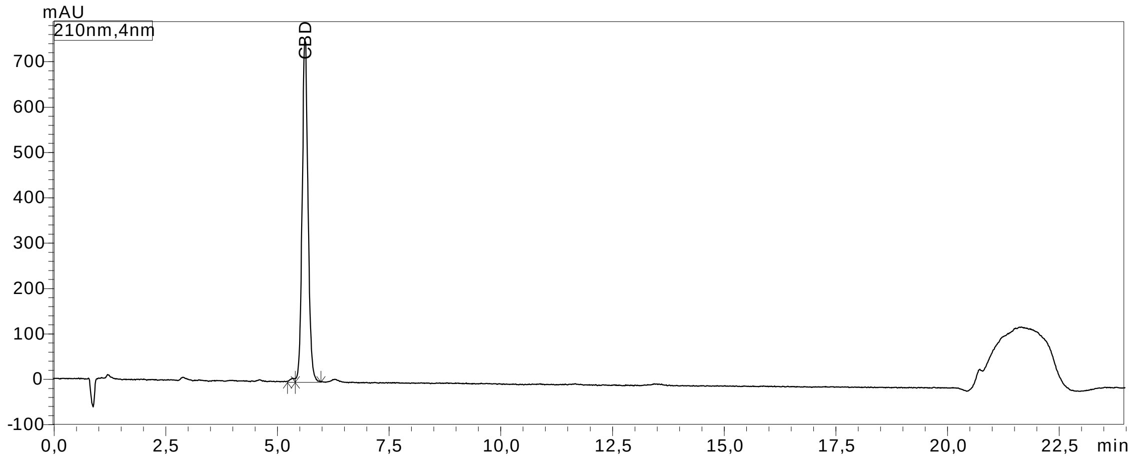

#11 – ENDOCA 300 mg CBD Hemp oil (3%) „heated”

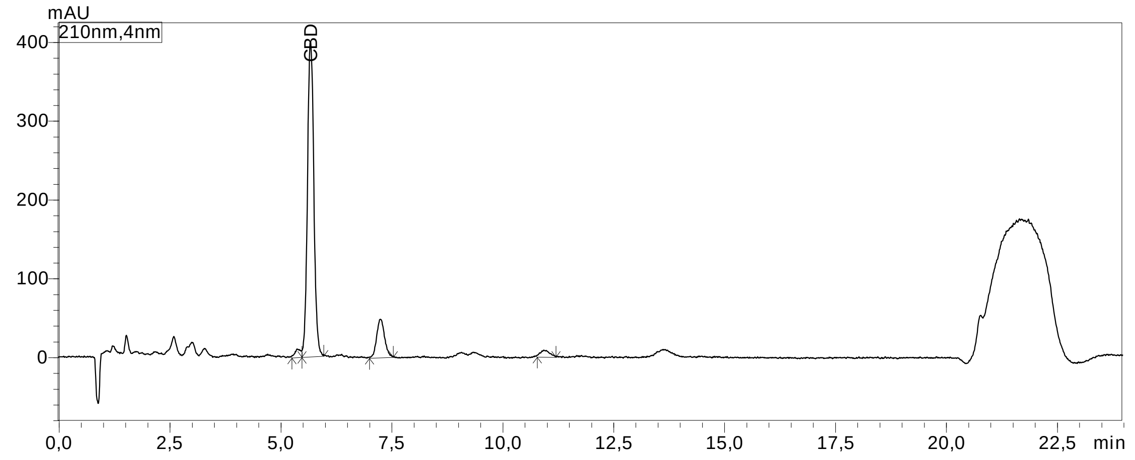

#12 BIOFORA HARMONY Premium 5% (500 MG) CBD OIL with Hemp oil 10 ML

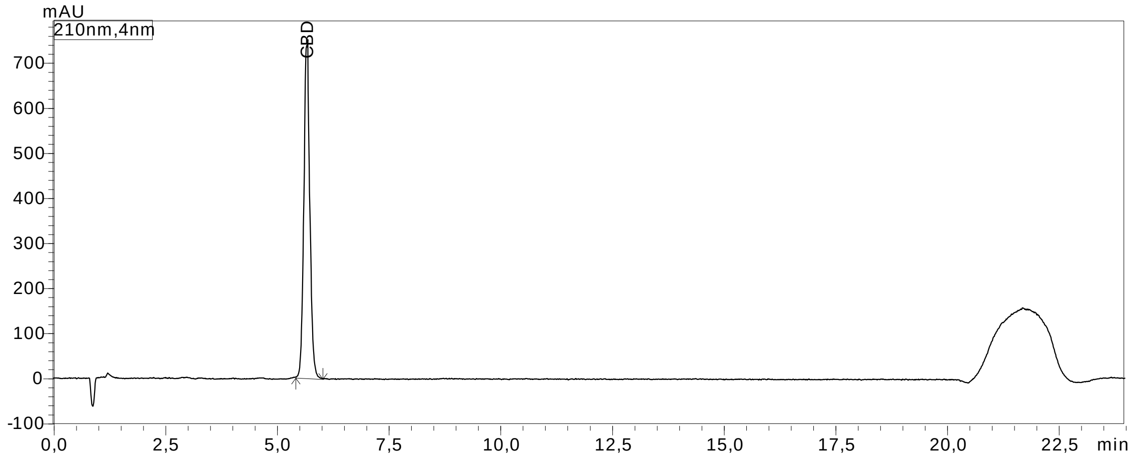

Calibration curve data

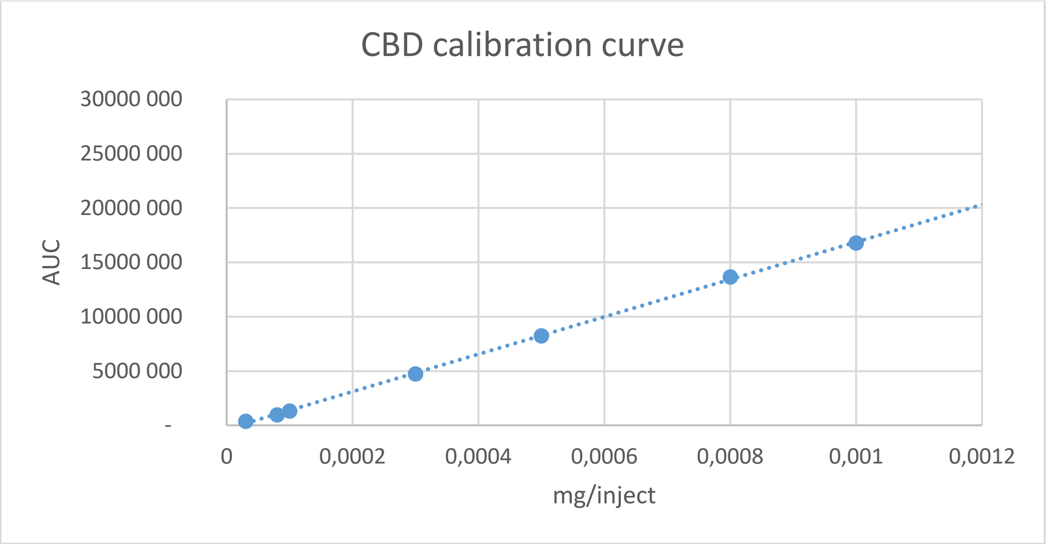

y = 17 205 893 410,0247x - 335 045,4975
R^2 = 0,9995
